# Prehospital arterial hypercapnia in acute heart failure is associated with admission to acute care units and emergency room length of stay: a retrospective cohort study

**DOI:** 10.1186/s12873-021-00411-9

**Published:** 2021-01-26

**Authors:** Mathias Fabre, Christophe A. Fehlmann, Birgit Gartner, Catherine G. Zimmermann-Ivoll, Florian Rey, François Sarasin, Laurent Suppan

**Affiliations:** 1 Division of Emergency, Department of Anaesthesiology, Clinical Pharmacology, Intensive Care and Emergency Medicine, Geneva University Hospitals and Faculty of Medicine, Rue Gabrielle-Perret-Gentil 4, CH-1205 Geneva, Switzerland; 2 Division of Medicine Laboratory, Department of Diagnostics, Geneva University Hospitals and Faculty of Medicine, Geneva, Switzerland; 3 Division of Cardiology, Department of medicine, Geneva University Hospitals and Faculty of Medicine, Geneva, Switzerland

**Keywords:** Acute heart failure, Arterial blood gas, Prehospital

## Abstract

**Background:**

Acute Heart Failure (AHF) is a common condition that often presents with acute respiratory distress and requires urgent medical evaluation and treatment. Arterial hypercapnia is common in AHF and has been associated with a higher rate of intubation and non-invasive ventilation in the Emergency Room (ER), but its prognostic value has never been studied in the prehospital setting.

**Methods:**

A retrospective study was performed on the charts of all patients taken care of by a physician-staffed prehospital mobile unit between June 2016 and September 2019 in Geneva. After approval by the ethics committee, charts were screened to identify all adult patients with a diagnosis of AHF in whom a prehospital arterial blood gas (ABG) sample was drawn. The main predictor was prehospital hypercapnia. The primary outcome was the admission rate in an acute care unit (ACU, composite of intensive care and high-dependency units). Secondary outcomes were ER length of stay (LOS), orientation from ER (intensive care unit, high-dependency unit, general ward, discharge home), intubation rate at 24 h, hospital LOS and hospital mortality.

**Results:**

A total of 106 patients with a diagnosis of AHF were analysed. Hypercapnia was found in 61 (58%) patients and vital signs were more severely altered in this group. The overall ACU admission rate was 48%, with a statistically significant difference between hypercapnic and non-hypercapnic patients (59% vs 33%, *p* = 0.009). ER LOS was shorter in hypercapnic patients (5.4 h vs 8.9 h, *p* = 0.016).

**Conclusions:**

There is a significant association between prehospital arterial hypercapnia, acute care unit admission, and ER LOS in AHF patients.

## Background

Acute Heart Failure (AHF) is a common but life-threatening condition with a high mortality. It often manifests by acute respiratory distress and requires urgent medical evaluation and management [1, 2]. Classical treatment includes use of vasodilators, diuretics and oxygen, as well as positive end-expiratory pressure (PEEP), with or without pressure support (PS), in case of severe AHF [3–11]. In the prehospital setting, application of PEEP, whether by use of continuous positive airway pressure (CPAP) or of noninvasive ventilation (NIV), is associated with an improvement in vital parameters and a reduction in both endotracheal intubation (ETI) and intensive care unit (ICU) admission rates [12–16]. Common negative prognostic markers for AHF include hypotension on admission, advanced age and polymorbidity, as well as an ischemic etiology [2].

Arterial blood gas (ABG) analysis has been shown to increase the quality of treatment in the prehospital setting [17]. Hypercapnia is a common finding in patients admitted to ICU because of AHF [18, 19]. Though Konishi et al. have shown that hypercapnia is associated with increased rates of both NIV and ETI in the intra-hospital setting [18], its predictive value in the prehospital environment is not known. The goal of this study was to evaluate the association between prehospital hypercapnia and admission to acute care units (ACU), including ICU and high-dependency units (HDU).

## Methods

### Study design

This was a retrospective cohort study, based on data obtained from an electronic charts review, exploring the association between hypercapnia in the prehospital field and admission to an ACU. The study protocol was approved by the institutional ethics committee of Geneva, Switzerland (project ID 2019–01559). Patient consent was waived by this committee.

### Setting

This study was carried out using data from the prehospital medical mobile unit (called SMUR for Service Mobile d’Urgence et de Réanimation) of the Geneva University Hospitals in Switzerland, the detailed organization of which has previously been described [12]. Briefly, the prehospital emergency medical response in Geneva consists of three levels of increasing expertise (advanced life support ambulance, medical mobile unit, senior emergency-medicine certified physician). The SMUR is staffed by a paramedic and a physician and is called upon according to specific dispatch protocols or if the first responding paramedics request it. Severe respiratory distress is among the criteria used to dispatch this unit. In addition to providing specific medical skills and knowledge, the SMUR can initiate NIV in the field well before arrival in the emergency room (ER). A Hamilton T1 ventilator (Hamilton Medical AG, Bonaduz, Switzerland) has been part of the standard SMUR equipment since 2013. ABG analysis has been possible since 2016 thanks to a portative i-STAT point-of-care blood analyzer (Abbott Point of Care inc., Princeton, New Jersey, USA). The indication for drawing an ABG sample in the field is left to the physician’s appreciation but is strongly suggested whenever NIV treatment is initiated. In order to avoid unnecessary delays, SMUR physicians are only allowed one single attempt on the radial artery in at most 1 min. There is no specific data available regarding the timing of ABG analysis, but SMUR physicians almost always begin by administering oxygen and often initiate NIV or other life-saving measure before drawing blood for analysis.

### Participants

Electronic charts of all patients for whom an intervention took place between 01.07.2016 and 30.09.2019 were screened for inclusion. There are only 126 different diagnoses that can be coded in the SMUR’s prehospital medical charts, and only two relate to AHF: “acute pulmonary edema” (APE) and “heart failure” (HF). All patients 18 years or older with any of these two diagnoses were initially included. Exclusion criteria were absence of an acute HF (lack of an acute component described in the report), cardiac arrest upon rescuers’ arrival, presence of a mixed or unclear diagnosis (such as a concomitant acute chronic obstructive pulmonary disease exacerbation), secondary transfer from another hospital or emergency structure, and limitation of care regarding ACU admission. Finally, patients for whom prehospital arterial ABG could not be obtained were also excluded.

### Data collection

Validated, indexed data of all the patients included in the analysis was imported to a REDCap (Vanderbilt University, Nashville, Tennessee, USA) electronic case report form (CRF). The first author (MF) was in charge of the full data collection, including all the manual data extraction required to complete the CRF. Any doubt was lifted by a consensus between MF and two of the other authors (CAF and LS).

### Exposure

The main predictor was prehospital hypercapnia, defined as PaCO_2_ equal or higher to 6 kPa (45 mmHg). According to our institutional guidelines, the accuracy of the i-STAT analyzer was regularly assessed by internal and external quality controls (QC). Internal QC were tested at multiple levels on each lot number at delivery for precision evaluation. External QC were performed at least 7 times per year for accuracy evaluation. QC of the ‘analyzer’ itself was achieved by use of an electronic check cartridge twice per year for electronics evaluation.

### Outcomes

The primary outcome was ACU admission, a composite outcome of admission to either HDU or ICU. Secondary outcomes were ER length of stay (LOS), orientation from ER (ICU, HDU, general and internal medicine wards, or discharge home), ETI (on field or in the first 24 h), hospital LOS and hospital mortality.

Week-end interventions were those performed on Saturdays and Sundays. Night interventions occurred between 7 PM and 7 AM. Length of intervention was the time elapsed between the arrival on site and the arrival at hospital. In-hospital ABG was retained if made in the first hour in emergency. Others blood samples were retained if realized in first 6 h in ER.

### Statistical analysis

As this was an exploratory study, no sample size calculation was performed. Groups were compared by Student’s *t*-test, the Mann-Whitney Wilcoxon test or the chi-squared test as appropriate. Results are expressed as mean ± standard deviation or absolute number and relative percentage. When appropriate, 95% confidence interval (95CI) are also reported. A *p*-value below 0.05 was considered significant. A single subgroup analysis was performed according to whether there was a correction of hypercapnia upon arrival in the ER. Missing data were reported as such and patients excluded from analyses if necessary (no imputation was made). Statistical analysis was performed using STATA version 14 (Stata Corporation, College Station, Texas, USA).

## Results

**Fig. 1:**
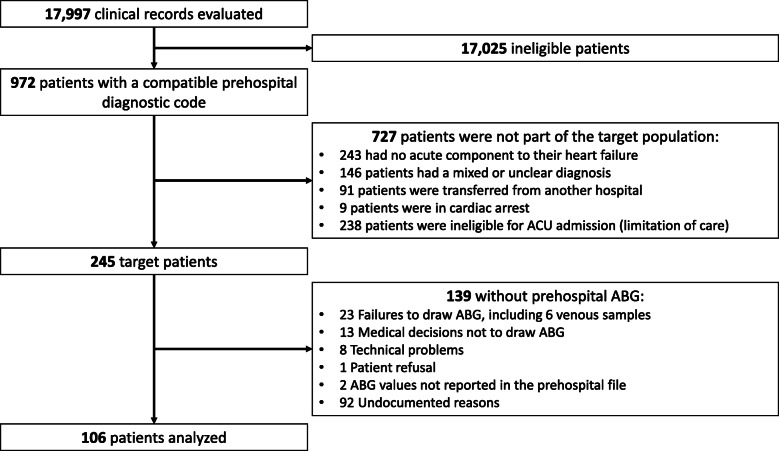
Study Flowchart

A total of 17′997 patients were taken care of by the SMUR during the study period, among whom 972 met the inclusion criteria. We then excluded 866 patients to finally include 106 patients (10.9%) in the analysis (Fig. 1).

Table 1 summarizes patients’ characteristics. Patients with (*n* = 61, 58%) and without (*n* = 45, 42%) hypercapnia were similar regarding personal history except for chronic hypertension and likely coronary heart disease and diabetes. Prehospital vital signs as well as Glasgow coma scale (GCS) were more severely altered in the hypercapnia group. Hypercapnic patients were more often put under NIV, which was set at significantly higher PS settings. Data were missing mainly for GCS (18%) and ER hypercapnia (19%).

**Table 1:** Patient’s characteristics^a^

	No prehospital hypercapnia (*n* = 45)	Prehospital Hypercapnia (*n* = 61)	*p*-value
Age (y)	79.2 ± 8.0	79.8 ± 8.1	0.719
Sex (f) – n (%)	21 (46.7)	37 (61.0)	0.153
Week-end intervention – n (%)	16 (36.4)	13 (22.4)	0.122
Night intervention – n (%)	26 (57.8)	37 (61.0)	0.765
Length of intervention (m)	41 ± 10	42 ± 12	0.701
**Personal history – n (%)**			
*Hypertension*	37 (82.2)	58 (95.1)	0.032
*Coronary heart disease*	19 (42.2)	17 (27.9)	0.123
*Atrial fibrillation*	22 (48.9)	23 (37.7)	0.250
*Pacemaker*	11 (24.4)	9 (14.8)	0.208
*Active cancer*	2 (4.4)	3 (4.9)	0.909
*Chronic obstructive pulmonary disease*	8 (17.8)	10 (16.4)	0.851
*Diabetes mellitus*	13 (28.9)	26 (42.6)	0.147
*Chronic renal failure*	18 (40.0)	29 (47.5)	0.480
*Prior hospitalisation for heart failure*	15 (33.3)	26 (42.6)	0.562
*Tobacco*	7 (15.6)	9 (14.8)	0.982
**Prehospital vital signs**			
*Heart rate (1/min)*	104 ± 20	114 ± 20	0.013
*Systolic blood pressure (mmHg)*	174 ± 31	189 ± 29	0.013
*Diastolic blood pressure (mmHg)*	94 ± 16	112 ± 18	< 0.001
*Respiratory rate (1/min)*	34 ± 9	38 ± 7	0.019
*Oxygen saturation (%)*	86 ± 9	83 ± 13	0.194
*Glasgow Coma Scale – n (%)*			0.018
*< 15*	3 (6.7)	17 (27.9)	
*15*	33 (73.3)	32 (52.5)	
*Missing values*	9 (20.0)	12 (19.7)	
Prehospital PaCO_2_ (kPa)	5.0 ± 0.6	7.7 ± 1.6	< 0.001
Prehospital pH	7.38 ± 0.06	7.23 ± 0.11	< 0.001
Prehospital NIV	34 (75.6)	59 (96.7)	0.001
*PEEP (mmHg)*	6.0 ± 1.7	6.4 ± 2.0	0.369
*PS*	8.1 ± 1.7	9.4 ± 2.1	0.006
**Hospital hypercapnia – n (%)**			0.001
*No hypercapnia*	29 (64.4)	24 (39.3)	
*Hypercapnia*	5 (11.1)	28 (45.9)	
*Missing value*	11 (24.4)	9 (14.8)	
**Emergency room work-up**			
*Haemoglobin (g/dl)*	130 ± 23	126 ± 20	0.225
*Serum creatinine (*μmol*/l)*	128 ± 75	152 ± 147	0.285
*Pro-B-type natriuretic peptide (ng/l)*	6225 ± 7548	6699 ± 9665	0.809

^a^ Plus–minus values are means ± standard deviation, Percentages may not total 100 due to rounding. PEEP: Positive End-Expiratory Pressure. PS: Pressure Support

The global ACU admission rate was 48% (51/106), with a significant difference between patients with and without hypercapnia (59.0% vs 33.3%, *p* = 0.009) (Table 2). This difference was likely due to a higher admission rate in ICU for hypercapnic patients. Hypercapnic patients who did not correct their PaCO_2_ upon ER arrival were not more likely to be admitted to an ACU than those who did correct their PaCO_2_ (respectively 71.4% versus 58%, *p* = 0.323). The change in PaCO_2_ in hypercapnic patients between prehospital and ER ABG was not associated with ACU admission either (respectively − 1.26 kPa for patients admitted to an ACU versus − 1.13 kPa for those who were not, *p* = 0.715). Emergency room LOS was significantly shorter in hypercapnic patients (5.4 ± 2.9 h versus 8.9 ± 9.0 h, *p* = 0.016). They also had higher intubation rate at 24 h, hospital LOS and mortality, albeit not significantly so.

**Table 2:** Outcomes^a^

	All (*n* = 106)	No prehospital hypercapnia (*n* = 45)	Prehospital Hypercapnia (*n* = 61)	*p*-value
**Primary outcome**				
ACU admission – n (%)	51 (48.1)	15 (33.3)	36 (59.0)	0.009
**Secondary outcomes**				
Emergency LOS (h)	6.9 ± 6.5	8.9 ± 9.0	5.4 ± 2.9	0.016
Orientation from ER – n (%)				0.003
*ICU admission*	25 (23.6)	4 (8.9)	21 (34.4)	
*HDU admission*	26 (24.5)	11 (24.4)	15 (24.6)	
*Ward*	51 (48.1)	26 (57.8)	25 (41.0)	
*Discharge directly from ER*	4 (3.8)	4 (8.9)	0 (0.0)	
ETI – n (%)	5 (4.7)	0 (0.0)	5 (8.2)	0.071
Hospital LOS (d)	13.1 ± 9.4	11.9 ± 6.8	14.1 ± 10.7	0.187
Hospital mortality – n (%)	5 (4.7)	1 (2.2)	4 (6.6)	0.392

^a^ Plus–minus values are means ± standard deviation, Percentages may not total 100 due to rounding

## Discussion

Our study shows a significant association between prehospital hypercapnia and ACU admission. This result seems mainly explained by the difference in ICU admissions between the two groups.

In our study, the prevalence of hypercapnia was higher than in most others (58% vs 33.7% in Konishi’s study [18], for example). To explain this difference, two hypotheses can be put forward. The first is related to the fact that we report prehospital values as opposed to other studies, which usually report in-hospital values. Therefore, an improvement consecutive to prehospital treatment before ER arrival cannot be ruled out in these settings. Indeed, our results show that almost 40% of patients with prehospital hypercapnia were normocapnic upon arrival in the ER. Another hypothesis is that prehospital physicians were more prone to performing ABG analyses in critically ill patients, thereby inducing a selection bias.

The difference between hypercapnic and non-hypercapnic patients regarding ACU admissions is probably best explained by the fact that hypercapnic patients seemed to be in a worse clinical state. These patients were indeed more tachycardic, more severely hypertensive, had lower GCS scores and higher respiratory rates. They also had a higher ETI rate at 24 h. The association between hypercapnia and worse clinical state is not fully elucidated, and the pathophysiological mechanisms leading to hypercapnia are not fully understood. It has been theorized that gas exchange might be impaired by the development of interstitial edema [20], and muscle fatigue, which leads to hypoventilation, certainly contributes to the increase in PaCO_2_ [21]. This latter effect probably best explains the link between hypercapnia and ETI, which was significant despite the limited sample size and the almost systematic use of NIV in hypercapnic patients. Indeed, prehospital NIV has been shown to significantly decrease the need for prehospital ETI [12].

In our study, hypercapnia was associated with a significantly lower LOS in the ER. Though it can hardly be assumed that hypercapnia alone was responsible for this time reduction in ER, one might argue that in-hospital orientation for patients with worse clinical state was more straightforward and could therefore account for this finding. Whether the ER physicians took prehospital ABG results into account could not be ascertained owing to the design of this study, and a prospective evaluation should be performed to determine the potential causality link. Should a link between early evaluation of ABG in the prehospital phase and ACU admission be proven, fast-tracks similar to those used for stroke or myocardial infarction could be considered [22, 23]. Such fast-tracks have been shown to shorten the delay to initiation of optimal in-hospital treatment, and might also decrease the strain on the ER and the waiting time of other patients [24].

Different limitations must be acknowledged. The small study sample did not have sufficient power to draw conclusions regarding mortality or to adjust our results for potential confounders. Moreover, potentially valuable data, such as usual medications, frailty parameters, or left ventricular function could not be obtained, further limiting the interpretation of our findings. Furthermore, though a clear relationship between ACU admission and prehospital hypercapnia has been found, such a relationship does not necessarily imply causality. One last limitation is the retrospective design of this study, as diagnosis of heart failure can differ from one physician to another. Nevertheless, as our prehospital chart only allows the use of a limited and standardized list of diagnoses, such differences might have been mitigated.

Despite these elements, the good quality of patient’s data can be highlighted, and is mainly linked to the review of electronically recorded charts the use of which lessens memorization bias. Another strong point is that, as prehospital hypercapnia has to our knowledge never been studied in AHF, this work shows new elements that could improve patient orientation in the ER. Prospective studies would be needed to better describe the role of prehospital hypercapnia in AHF.

## Conclusion

In conclusion, this study shows a significant association between prehospital arterial hypercapnia, ACU admissions, ER LOS and ETI in patients with AHF. Prospective studies should now be carried out to confirm these results before prehospital ABG can be recommended on a routine.

## Data Availability

The datasets used and analysed during the current study are available from the corresponding author on reasonable request.

## Abbreviations

ABG: Arterial Blood Gas
ACU: Acute Care Unit
AHF: Acute Heart Failure
APE: Acute pulmonary Edema
CRF: Case Report Form
CPAP: Continuous Positive Airway Pressure
ER: Emergency Room
ETI: Endotracheal Intubation
GCS: Glasgow Coma Scale
HDU: High-dependency Unit
HF: Heart Failure
ICU: Intensive Care Unit
LOS: Length of Stay
NIV: Non-Invasive Ventilation
PEEP: Positive End-Expiratory Pressure
PS: Pressure Support
QC: Quality Control SMUR Service Mobile d’Urgences et de Réanimation
